# Nelfinavir and Nelfinavir Analogs Block Site-2 Protease Cleavage to Inhibit Castration-Resistant Prostate Cancer

**DOI:** 10.1038/srep09698

**Published:** 2015-04-16

**Authors:** Min Guan, Leila Su, Yate-Ching Yuan, Haiqing Li, Warren A. Chow

**Affiliations:** 1 Department of Molecular Pharmacology, Beckman Research Institute of the City of Hope, Duarte, CA, USA; 2 Department of Molecular Medicine, Beckman Research Institute of the City of Hope, Duarte, CA, USA; 3 Department of Medical Oncology and Therapeutics Research, City of Hope, Duarte, CA, USA

## Abstract

Nelfinavir and its analogs inhibit proliferation and induce apoptosis of castration-resistant prostate cancer through inhibition of site-2 protease (S2P) activity, which leads to suppression of regulated intramembrane proteolysis. Western blotting in nelfinavir and its analog treated cells confirms accumulation of precursor SREBP-1 and ATF6. Nelfinavir and its analogs inhibit human homolog *M. jannaschii* S2P cleavage of an artificial protein substrate CED-9 in an *in vitro* proteolysis assay in a dose-dependent manner. Nelfinavir and its analogs are more potent inhibitors of S2P cleavage activity than 1,10-phenanthroline, a metalloprotease-specific inhibitor. Further, cluster analysis of gene expression from treated DU145 and PC3 cell lines demonstrate a close similarity of nelfinavir, its analogs, and 1,10-phenanthroline. These results show nelfinavir and its analogs inhibit castration-resistant prostate cancer proliferation by blocking regulated intramembrane proteolysis through suppression of S2P cleavage activity. This leads to accumulation of precursor SREBP-1 and ATF6, and development of insufficient reserves of their transcriptionally-active forms. The present results validate S2P and regulated intramembrane proteolysis as novel therapeutic targets for castration-resistant prostate cancer therapeutics. A clinical trial of nelfinavir or its analogs should be developed for castration-resistant prostate cancer.

Castration-resistant prostate cancer (CRPC) generally develops in hormone-sensitive prostate cancer (HSPC) after 13–24 months of androgen-deprivation therapy^1^. After progression, the median overall survival for men with metastatic CRPC is 15–18 months^2^,^3^. CRPC demonstrates androgen receptor (AR)-dependent pathway reactivation due to AR overexpression, AR mutation, and AR activation^4^.

Development of a “lipogenic phenotype” is a complementary path to CRPC independent of AR reactivation. Here, increased de novo fatty acid (FA) synthesis occurs as a consequence of increased expression of lipogenic genes in CRPC^5^. The FAs are used by cancer cells to produce lipids for membrane synthesis, β-oxidation for energy production, and lipid-based post-translational modification. Sterol regulatory element-binding proteins (SREBPs) regulate both cholesterol synthesis and lipogenesis^6^. SREBP-1a and -1c governs lipogenesis by transcriptional regulation of fatty acid synthase (FAS)^7^. FAS is a key enzyme required for the synthesis of long-chain FAs from acetyl-coenzyme A (CoA). SREBPs are produced as inactive precursors bound to the endoplasmic reticulum (ER) by SREBP cleavage-activating protein (SCAP)^8^,^9^. SCAP binds insulin-induced gene-1 or -2 (Insig-1 or -2) in the ER^10^. Insigs anchor the SREBP-SCAP complex to the ER; during periods of cholesterol or FA depletion, SCAP and Insigs fail to interact, and the precursor complex is transported to the Golgi, where it is processed in two sequential cleavage steps by serine protease, Site-1 (S1P), and metalloprotease, Site-2 proteases (S2P), to release the mature, transcriptionally-active, amino-terminal SREBP into the nucleus; there, it forms a dimer and binds to the promoter of target genes like FAS. This integrated process is known as Regulated Intramembrane Proteolysis (RIP)^11^,^12^,^13^,^14^. RIP is also necessary for post-translational processing of activating transcription factor 6 (ATF6), which is necessary to mediate an integrated unfolded protein response (UPR) in response to ER stress that develops from ER protein misfolding^15^.

Nelfinavir, an HIV protease inhibitor (PI) used in combination antiretroviral therapy, also demonstrates unique properties as a novel anticancer agent^16^. It inhibits Akt phosphorylation, signal transducer and activation of transcription factor 3 (STAT3) signaling, cyclin-dependent kinase 2 (CDK2) function, heat shock protein 90 (HSP90) function, and general kinase activity^17^,^18^,^19^,^20^,^21^,^22^,^23^. Notably, nelfinavir also downregulates and blocks AR signaling in hormone-sensitive prostate cancer cells^20^. Despite extensive studies on the anticancer activity of nelfinavir, the precise underlying molecular mechanism remains uncertain. We have shown that nelfinavir inhibits RIP-mediated activation of SREBP-1 and ATF6 in CRPC as either siRNA-mediated knockdown of S2P or metalloprotease inhibitor-mediated S2P inhibition blocked nuclear translocation of green fluorescence-labeled SREBP-1 and ATF6^24^. In the current study, we definitively demonstrate that nelfinavir blocks S2P cleavage activity in CRPC to inhibit proliferation and induce apoptosis *in vitro*. Further, we identify several structural analogs of nelfinavir which possess similar biological function. Some of these analogs possess a genome-wide gene signature response to treatment similar to nelfinavir. These results can further the development of this class of agents for anticancer therapeutics.

## Results

### Screening of NCI Open Chemical Repository Collection and identification of compounds

**Figure 1:**
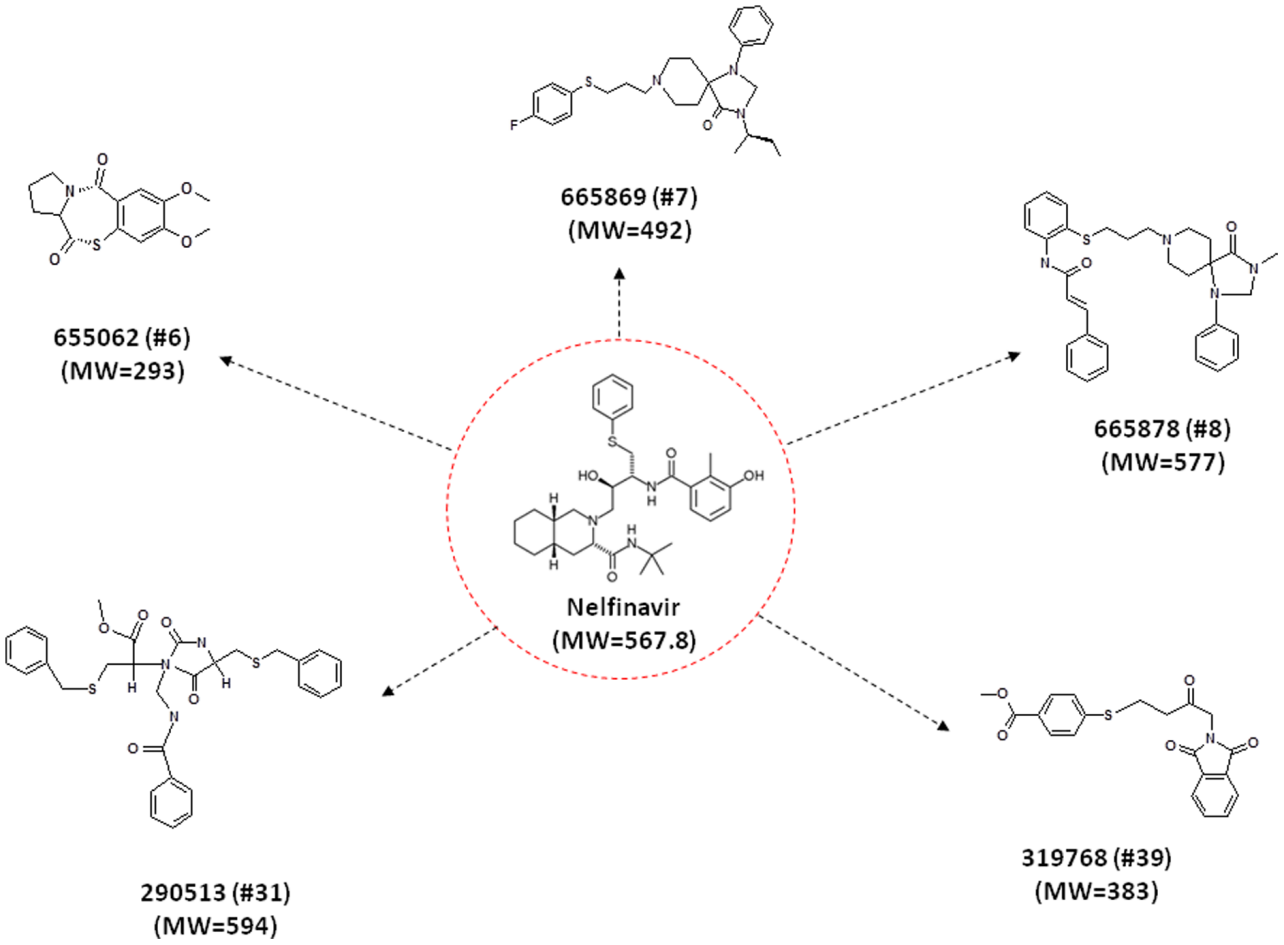
Structure of nelfinavir and its analogs. A search of the NCI Compound Library with >50% similarity to Nelfinavir (Tanimoto coefficient = 0.50) was performed with SYBYL-X software (Tripos, St. Louis, MO). 231 compounds were identified and subsequently clustered into 16 groups by structure. The structure and molecular weight (MW) of nelfinavir and its analog #6, 7, 8, 31, 39, are shown here.

We and others previously reported nelfinavir has unique anti-cancer activity^16^,^17^,^18^,^19^,^20^,^21^,^22^,^23^,^24^. The NCI Open Chemical Repository Collection has 250,251 compounds. A search of the NCI Chemical Repository Collection with >50% similarity to nelfinavir (Tanimoto coefficient ≥ 0.50) was performed with SYBYL (Tripos-Certara, St. Louis, MO). 231 compounds were identified and subsequently clustered into 16 groups by structure. A hit list of 80 compounds was generated, and 48 were available for actual testing. Figure 1 shows the structure of nelfinavir and analog #6, 7, 8, 31, 39, in which the present data was generated.

### Nelfinavir analogs inhibit proliferation and induce apoptosis in CRPC

**Figure 2:**
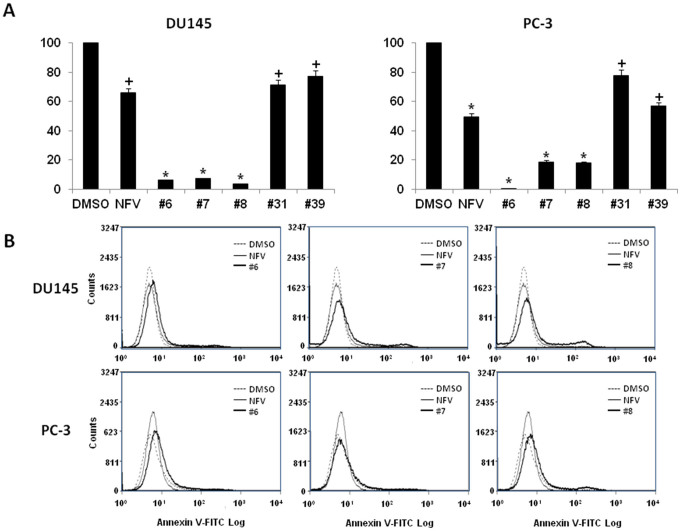
Nelfinavir analogs inhibit proliferation and induce apoptosis in CRPC. (A) Nelfinavir analogs inhibit CRPC proliferation. DIMSCAN assay was performed in nelfinavir- or analog #6, 7, 8, 31, 39-treated DU145 and PC-3 cells. 1000 cells were seeded in a 96-well plate for overnight incubation followed by treatment with DMSO, nelfinavir or its analogs (10 μM) for 3 days. Each experiment was performed in triplicate. Results are presented as mean ± SD. *p < 0.01, +*p* < 0.05 compared to DMSO control. (B) Nelfinavir analogs induce CRPC apoptosis. DU145 and PC-3 cells were treated with DMSO or 10 μM of nelfinavir or analog #6, 7, 8, 31, 39. After 24 hr, cells were harvested and stained with Annexin V-FITC for detection of apoptosis. Image represents three repeated experiments.

DU145 and PC-3 cells were treated with 10 μM NFV and the available analogs for 72 hr prior to assaying for proliferation using the DIMSCAN system. As shown in Figure 2A, proliferation was significantly reduced in analogs #6, #7 and #8 treated CRPC cells comparing to nelfinavir treated cells. Only moderate effects were observed in CRPC cells treated with analogs #31 and #39 compared to nelfinavir. Results for analogs less potent than nelfinavir are not shown. Moreover, increased apoptosis was detected in CRPC cells by FACS detection of FITC-annexin V treated with analogs #6, #7 and #8 compared to nelfinavir-treated CRPC cells (Figure 2B). Both the proliferation and apoptosis assays suggest analogs #6, #7 and #8 are more potent than nelfinavir at equimolar concentrations.

### Nelfinavir analogs increase precursor SREBP-1 and ATF6 protein accumulation

**Figure 3:**
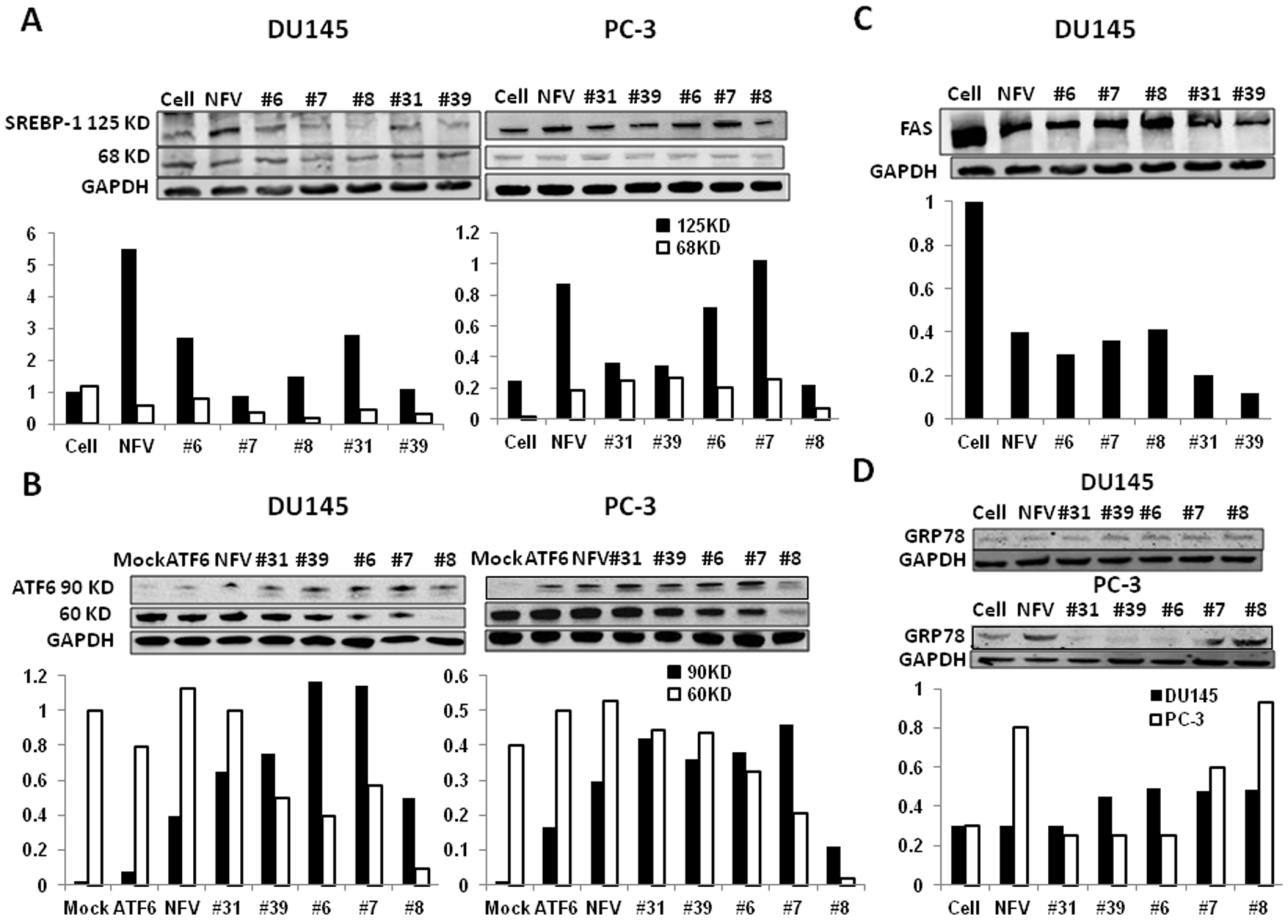
Nelfinavir and its analogs increase precursor SREBP-1 and ATF6 protein accumulation, and decrease FAS and increase GRP78 expression. DU145 and PC-3 cells were treated with nelfinavir or analogs (10 μM) for 24 hr and lysate was harvested for Western blot analysis of SREBP-1, FAS and GRP78. pATF6-EGFP (or mock) was transfected 24 hr prior to the treatment of nelfinavir or analogs for 24 hr for analysis of ATF6. (A–B) Nelfinavir analogs increase precursor SREBP-1 and ATF6 accumulation. (C) Nelfinavir analogs decrease FAS expression. (D) Nelfinavir increases GRP78 expression. The images are representative of at least three experiments. Cropped blots are from gels run under same condition. Quantification of Western blot was by Quantity One (Bio-Rad, Hercules, CA) and normalized to control GAPGH.

Our previous data showed that nelfinavir inhibited CRPC proliferation through inhibition of RIP-mediated processing of precursor SREBP-1 and ATF6^24^. Accordingly, both precursor and mature SREBP-1 and ATF6 detection (Fig. 3 A and 3B) were quantified by Western blot in CRPC cells treated with nelfinavir or its analogs. As shown in Fig. 3A and B, nelfinavir and and all five analogs increase the precursor level of ATF6 whereas only nelfinavir, #6, #31 increase SREBP-1 precursor in DU145 cells. Nelfinavir, and all analogs with the exception of #8 increase detection of precursor SREBP-1 and ATF6 in PC-3 cells. As a transcriptional target of SREBP-1, FAS expression was examined in nelfinavir and nelfinavir analog-treated DU145 cells. The immunoblot demonstrates reduced FAS expression (Fig. 3C).

To evaluate ER stress induced by nelfinavir, GRP78 level was detected by immunoblotting. GRP78 is an endoplasmic reticulum (ER) chaperone that binds newly synthesized proteins as they translocate to the ER (27). Its production is markedly induced under conditions that lead to the accumulation of unfolded polypeptides in the ER (ER stress). An increase in GRP78 was observed in CRPC cells treated with nelfinavir and analogs #7 and #8 (Fig. 3D); this indicates nelfinavir and its analogs generate significant ER stress.

### Nelfinavir analogs inhibit *mj*S2P cleavage of CED-9

**Figure 4:**
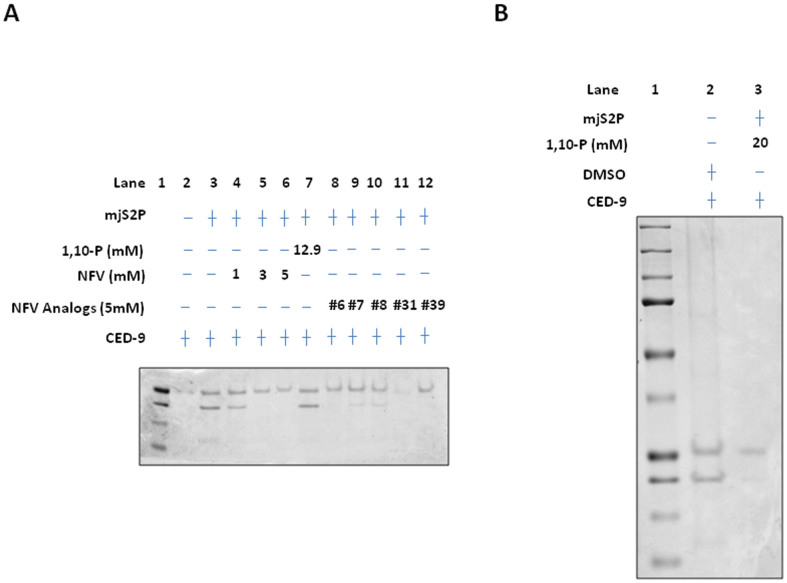
Nelfinavir and its analogs inhibit mjS2P cleavage of CED-9. (A) Nelfinavir inhibits mjS2P cleavage of CED-9. The proteolytic activity of mjS2P was examined by an *in vitro* cleavage assay: CED-9 (1 mg/ml) and mjS2P (0.1 mg/ml) were co-incubated at room temperature for 30 minutes; for the inhibition assays, 1,10-phenanthroline, nelfinavir or analog #6, 7, 8, 31, 39 was incubated with mjS2P at indicated concentration for 5 minutes at room temperature prior to addition of CED-9. The reaction was stopped by SDS sample buffer and the cleavage products were analyzed by SDS-PAGE and Coomassie staining. Cropped blots are from gels run under same condition. (B) High dose (20 mM) 1,10-phenanthroline completely inhibits mjS2P cleavage of CED-9. DMSO served as a negative control.

Shi, et al. reconstituted an *in vitro* proteolysis assay, in which the transmembrane core domain (residues 1 to 224) of the S2P homolog *mj*S2P cleaved an artificial protein substrate CED-9 in detergent micelles^25^. As shown in Fig. 4A lane 3, *mj*S2P efficiently cleaves its substrate CED-9. This substrate cleavage can be inhibited in a concentration-dependent manner by 1,10-phenanthroline, a metalloprotease-specific inhibitor (Fig. 4A lane 7 and 4B lane 3). Notably, substrate cleavage inhibition was similarly observed for nelfinavir in a dose-dependent manner (Fig. 4A lane 4–6). Strikingly, at the same concentration [5 mM], nelfinavir, analogs #6 and #39 achieve complete inhibition of substrate cleavage equivalent to 1,10-phenanthroline [20 mM] (Fig. 4A lanes 6, 8, 12 and 4B lane 3), which clearly suggests nelfinavir and its analogs are more potent inhibitors of S2P cleavage activity than 1,10-phenanthroline. These data strongly support the hypothesis that nelfinavir and its analogs produce significant ER stress in CRPC. This results from direct inhibition of S2P-mediated RIP processing of SREBP-1 and ATF6, which results in accumulation of their unfolded, precursor forms. These results demonstrate that nelfinavir is a more potent inhibitor of CED-9 cleavage than 1,10-phenanthroline.

### Cluster analysis of RNA sequencing by nelfinavir and its analogs

**Figure 5:**
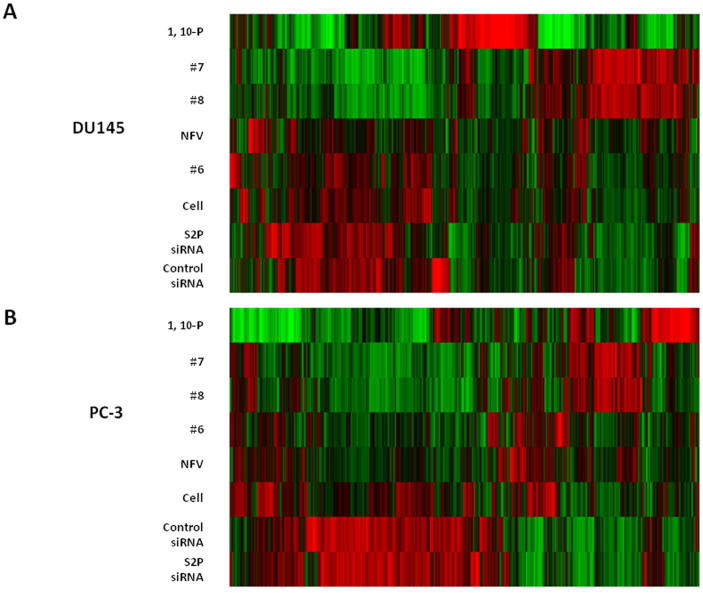
Cluster analysis of RNA sequencing by nelfinavir and its analogs. Gene expression profiles generated in DU145 (A) and PC-3 (B) cells treated with nelfinavir, its analogs, or 1,10-phenanthroline were analyzed by next generation sequencing. S2P siRNA or control siRNA transfected cells were analyzed as well. Maximum expression < 0.01RPKM was used to filter and exclude very low expressing genes. Each sample was compared with untreated, control cells using +/− 1.5 fold change as the minimum threshold.

Gene expression profiles of CRPC DU145 and PC-3 cells generated after treatment with nelfinavir or its analogs were analyzed by next generation RNA sequencing. Maximum expression < 0.01RPKM was used to filter and exclude very low expressing genes. Each sample was compared with untreated, control cells using +/− 1.5 fold change as the minimum threshold. RNA sequencing alignment was performed with TopHat v1.2. QC stats, mRNA quantification, differential expression and GO analysis were performed with Partek'sRNA-SEQ workflow^26^. Network and pathway analysis use Ingenuity Pathway Analysis (IPA). Although moderately different clustering profiles on hierarchical clustering are noted between DU145 and PC-3 cells (Fig. 5), analogs #7 and #8 most similarly reproduced the expression profile for 1, 10-phenanthroline. Among the analogs, #6 clustered most closely to nefinavir. In PC-3 cells (Fig. 5) all the analogs generated expression profiles similar to nelfinavir and 1, 10- phenanthroline; this supports the hypothesis that these analogs similarly inhibit S2P cleavage in CRPC. In contrast, the distinct gene expression profiles generated by gene knockdown through S2P siRNA from both of the cell lines suggest the possibility of residual S2P catalytic activity due to inadequate S2P knockdown, alternative mechanisms of S2P inhibition between the small molecule inhibitors and siRNA, or possible off-target effects of small molecule S2P inhibitors.

### Nelfinavir induces gene expression of S2P substrates and their targets

**Figure 6:**
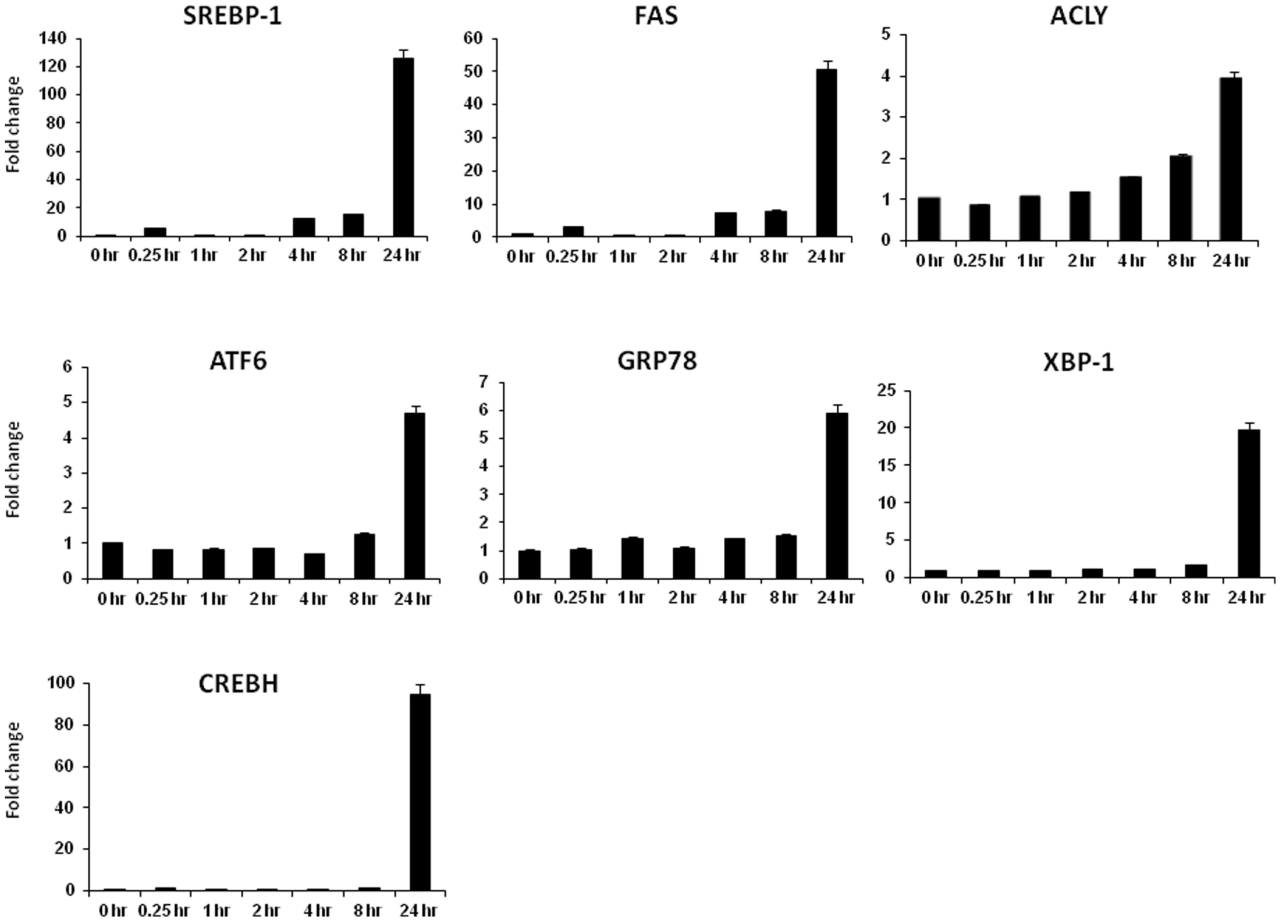
Nelfinavir induces S2P target gene expression. Nelfinavir (10 μM) treated DU145 cells were harvested at 0, 0.25, 1, 2, 4, 8, 24 hr to extract total RNA to examine S2P target (SREBP-1, ATF6 and CREBH) gene and their downstream (FAS, ACLY, GRP78 and XBP-1) gene expression by quantitative RT-PCR.

Nelfinavir (10 μM)-treated DU145 cells were harvested at serial times points, and total RNA was extracted to examine gene expression of S2P substrates and their targets by quantitative RT-PCR. SREBP-1, ATF6 and CREBH are cleaved by S2P in RIP, and were examined. Additionally, their respective downstream target genes: *FAS*, *ACLY* and *GRP78* were examined, as well as the UPR gene, *XBP-1*. As shown in Figure 6, biphasic expression in some genes was observed at early induction (0–4 hr), however, a time-dependent increase was observed at late induction (4–24 hr). Quite notably, there is a significant increase of gene expression by 24 hr of nelfinavir treatment. Extended incubation indicates a peak expression was achieved at 48 hr in most of genes with the exception of SREBP-1 at 72 hr post induction (Supplementary Figure 1).

## Discussion

Our previous data suggested that S2P is the primary target of nelfinavir because inhibition of S2P by siRNA or 1,10-phenanthroline reproduced the nelfinavir-treated CRPC phenotype *in vitro*^24^. Nelfinavir-mediated inhibition of S2P proteolytic activity leads to inhibition of RIP and accumulation of unprocessed, precursor SREBP-1 and ATF6. Those further induce ER stress and a faulty UPR resulting from insufficient transcriptionally-active ATF6 leads to caspase-dependent apoptosis. Nelfinavir clearly is a multi-targeted drug. It blocks AR signaling and downregulates the AR^20^; it depletes transcriptionally-active SREBP-1 thereby reducing FAS expression to inhibit the “lipogenic phenotype;” and it induces ER stress and apoptosis.

Our current data demonstrate that nelfinavir and its analogs inhibit *mj*S2P-mediated proteolysis of an artificial protein substrate CED-9 *in vitro*. *mj*S2P was used because repeated attempts to express human S2P in human embryonic kidney (HEK) 293 cells proved unsuccessful, likely due to the inherent difficulties of working with multipass membrane proteins^27^. Because BCL-2 is the human homolog of *C. elegans* CED-9, it was used as an alternative substrate in the *in vitro* *mj*S2P cleavage assay. Similar inhibition of BCL-2 cleavage by *mj*S2P with nelfinavir was observed (data not shown), which supports our hypothesis that nelfinavir inhibits S2P to interfere RIP in CRPC. Interestingly, other HIV PIs block the zinc metalloproteinase ZMPSTE24 and lead to accumulation of prelamin A in cells^28^. S2P, like ZMPSTE24, is an integral membrane metalloprotease of the ER^29^. Notably, it was hypothesized that HIV PIs would also inhibit S2P and would account for the changes in lipid metabolism in patients taking HIV PIs because of the effects on SREBP metabolism^29^.

In mammalian cells, S2P is essential due to its role in the production of unsaturated fatty acids and cholesterol through activation of SREBPs; in the absence of exogenous lipid, cells lacking S2P cannot survive^13^,^30^. S2P is also important in the endoplasmic reticulum (ER) stress response, activating several different membrane-bound transcription factors. All known substrates for intramembrane S2P are membrane-bound transcription factors. Cleavage of those precursors by S2P releases a cytoplasmic domain that travels to the nucleus to mediate transcriptional activation of target genes. Humans harboring reduction-of-function mutations in S2P exhibit an array of pathologies ranging from skin defects to neurological abnormalities^30^.

24 hour treatment of DU145 cells with nelfinavir or analogs led to reduced detection of FAS by immunoblotting (Fig. 3C). This is consistent with reduced available nuclear SREBP-1 due to inhibition of S2P (Fig. 3A). In contrast, qRT-PCR showed minimal change in relative expression of *SREBP-1* nor its target genes, *FAS* and *ACLY* until 24 hours of treatment, whereupon all three genes are induced (Fig. 6). We postulate, once *FAS* is reduced and intracellular levels of cholesterol and fatty acid are depleted, the cholesterol-sensing function of *SREBP-1* signals to increase *SREBP-1* transcription. *SREBP-1* induces its own transcriptional activation due to the presence of SRE binding sites within the *SREBP-1* promoter in a feed-forward, amplification system^31^. Also, the limited half-life of nelfinavir likely also contributes. We believe this accounts for the seemingly discordant results of the *FAS* gene and protein expression data. These gene transcription results are consistent with the fold-change in gene expression analysis by RNA sequencing (data not shown). Our data support the hypothesis that nelfinavir targets S2P catalysis downstream gene expression to regulate CRPC metabolism.

Screening of the NCI Chemical Repository Collection offers an effective way to identify potentially active compounds and rapidly move candidate drugs into the clinic. The NCI database of 250,251 compounds was scanned, and 231 compounds were identified with >50% similarity to nelfinavir and M8. The 231 compounds were clustered into 16 groups by their structure features and a hit list of 80 compounds was generated by visual inspection and analysis. Here we identified three analogs (#6, 7 and 8) which inhibit CRPC proliferation more potently than nelfinavir. Most importantly, these analogs possess properties similar to nelfinavir, including: induction of apoptosis; accumulation of SREBP-1 and ATF6 precursors; and notably, suppression of RIP through S2P inhibition. Further studies to optimize the potency of nelfinavir analogs should be done based on structure-activity relationship (SAR) analysis.

Our study demonstrates that nelfinavir and its analogs inhibit CRPC proliferation through direct inhibition of S2P cleavage and RIP processing. This leads to accumulation of precursor SREBP-1 and ATF6, and development of insufficient reserves of their transcriptionally-active forms. The present results demonstrate a novel strategy targeting S2P and RIP for cancer therapeutics. Further, they form the basis for development of clinical trials of nelfinavir and its analogs for CRPC.

## Methods

### Cell culture

DU145 and PC-3 CRPC cell lines were purchased from the American Type Culture Collection (Manassas, Virginia) and maintained in MEM and F-12K (Life Technologies, INC., Logan, UT) with 10% fetal bovine serum.

### Chemicals and reagents

Nelfinavir and its analogs were obtained from the NIH AIDS Research & Reference Reagent Program (Germantown, MD) and Developmental Therapeutics Program, Division of Cancer Treatment and Diagnosis, National Cancer Institute, NIH (Bethesda, MD). Nelfinavir and its analogs were reconstituted in dimethyl sulfoxide (DMSO). 1,10-phenanthroline was purchased from Sigma-Aldrich (St. Louis, MO). An S2P homolog protein from *M. jannaschii* (*mj*S2P) was produced in E. coli from the pET15b-mjS2P expression plasmid by Profacgen Inc. (Shirley, NY). This plasmid encodes the transmembrane core domain of *mj*S2P (resides 1–224), and was a kind gift from Dr. Yigong Shi (Princeton University, Princeton, NJ)^25^. The BCL-2 homolog *C. elegans* protein 9 (CED-9) was a kind gift from Dr. Ding Xue (University of Colorado, Boulder, CO). SREBP-1 and GRP78 antibodies were purchased from Santa Cruz Biotechnology, Inc. (Santa Cruz, CA) and ATF6 antibody was purchased from Abcam, Inc. (Cambridge, MA).

### Apoptosis and proliferation assay

DU145 and PC-3 cells were treated with nelfinavir or its analogs for 24 hr, and collected for detection of annexin V-positive apoptotic cells (Santa Cruz Biotechnology, Inc.) by flow cytometry. Similarly, proliferation in cells treated with nelfinavir or its analogs for 72 hr was determined by DIMSCAN (Bioimaging Solutions Inc., San Diego, CA) as previously described^24^.

### Western blot analysis

CRPC cells were treated with 10 μM nelfinavir, analogs, or DMSO for 24 hr, and cell lysates were collected to detect SREBP-1 and GRP78 expression by Western blot. pCGN-ATF6, a kind gift from Dr. Amy Lee, (University of Southern California, Los Angeles, CA) was transfected into cells 24 hr prior to treatment with nelfinavir and analogs for ATF6 detection.

### S2P cleavage assay

The proteolytic activity of *M. jannaschii* S2P (*mj*S2P) was examined by an *in vitro* cleavage assay^25^. The membrane-associated protein CED-9 was used as an artificial protein substrate for *mj*S2P. The assay was performed for 30 minutes in a buffer containing 100 mM sodium chloride, 0.2% (w/v) DM, and 10 mM Tris-Cl, pH 8.0. The concentrations of the substrate protein and mjS2P were approximately 1 and 0.1 mg/ml. For the inhibition assays, nelfinavir, its analogs, or 1,10-phenanthroline were incubated with the *mj*S2P protease for 5 minutes prior to addition of substrate. The reaction was stopped with SDS sample buffer, and the resulting cleavage products were analyzed by SDS-PAGE and Coomassie staining.

### RNA sequencing and data analysis

DU145 and PC-3 cells were treated with 10 μM of nelfinavir, its analogs, or 20–40 μM of 1,10-phenanthroline for four hours, and harvested to extract total RNA. Additionally, DU145 and PC-3 cells were transfected with S2P siRNA for 48 hours, and collected for RNA extraction. RNA sequencing was performed by Illumina next-generation sequencing by the City of Hope Integrative Genomics Core (Duarte, CA), and the resulting gene expression data was analyzed by the City of Hope Bioinformatics Core.

### Quantitative RT-PCR

Quantitative reverse transcriptional PCR (q-RT-PCR) was carried out in total RNA extracted from nelfinavir-treated PC-3 and DU145 cells. Relative primers are shown in Supplementary data. Relative gene-expression quantification method was used to calculate the fold change of mRNA expression.

### Statistical Analysis

Data were presented as the mean ± SD of three independent experiments. Group comparisons for continuous data were done with student's t-test for independent means or one-way ANOVA.

## Supplementary Material

Supplementary Information Supplementary Information
