# Supplementary material for: Nelfinavir and Nelfinavir Analogs Block Site-2 Protease Cleavage to Inhibit Castration-Resistant Prostate Cancer
Source: Sci Rep. 2015 Apr 16;5:9698. doi: 10.1038/srep09698 (PMC4816264; doi:10.1038/srep09698)
Supplement: Supplementary Information [file srep09698-s1.pdf]

Nelfinavir and Nelfinavir Analogs Block Site-2 Protease Cleavage to Inhibit Castration-Resistant Prostate Cancer

Min Guan[1], Leila Su[2], Yate-Ching Yuan[2], Haiqing Li[2], Warren A. Chow[1, 3]*

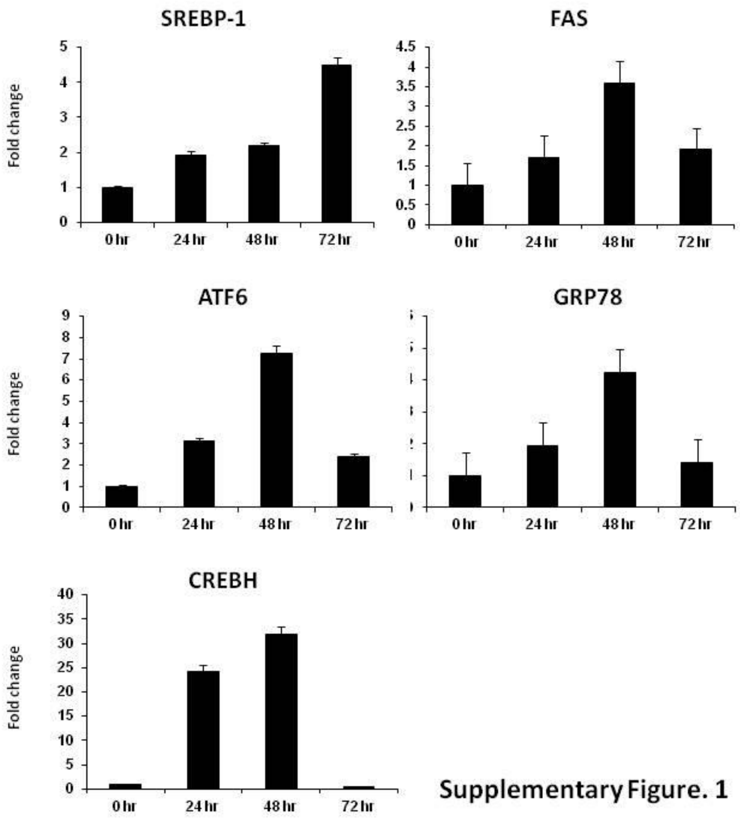

Supplementary Figure 1. Nelfinavir induces S2P target gene expression. Nelfinavir (10 µM) treated DU145 cells were harvested at 0, 24, 48 and 72 hr to extract total RNA to examine gene expression of direct S2P substrates (SREBP-1, ATF6 and CREBH) and their downstream gene targets (FAS, ACLY, GRP78 and XBP-1) by quantitative RT-PCR.

RT-PCR primer sequences:
SREBP-1 5'-gtaaggaagagtcagtgccactgagcat 3'-tatagcctaacacaggggtggagctgaa
FASN 5'-attgaagtttacaaggagctgcgtctg 3'-taccatgaagctcacccagttatcctt
ACLY 5'-taacttggtagtcaagccagaccagctg 3'-aagtggccttgccaactgtggcttcctg
ATF6 5'-aatgagctgcaattggaagcagcaaatg 3'-cgaactgtacagatttggttgttgatgt
GRP78 5'-tcacaaagacatttgctcctgaagaaat 3'-tattaaaataggctggtacagtaacaac
XBP-1 5'-ttaaggcgctgaggaggaaactgaaaaa 3'-ggaaagtttttggttctcttcttctaaa
CREB 5'-tatgctcagaatcaggagttacagagga 3'-aatgtctgggctgacttgctggtggact
